# Precision Medicine and the Role of Biomarkers of Radiotherapy Response in Breast Cancer

**DOI:** 10.3389/fonc.2020.00628

**Published:** 2020-04-24

**Authors:** James Meehan, Mark Gray, Carlos Martínez-Pérez, Charlene Kay, Lisa Y. Pang, Jennifer A. Fraser, Amy V. Poole, Ian H. Kunkler, Simon P. Langdon, David Argyle, Arran K. Turnbull

**Affiliations:** ^1^Translational Oncology Research Group, Institute of Genetics and Molecular Medicine, Western General Hospital, University of Edinburgh, Edinburgh, United Kingdom; ^2^The Royal (Dick) School of Veterinary Studies and Roslin Institute, University of Edinburgh, Edinburgh, United Kingdom; ^3^Breast Cancer Now Edinburgh Research Team, Institute of Genetics and Molecular Medicine, Western General Hospital, University of Edinburgh, Edinburgh, United Kingdom; ^4^School of Applied Science, Sighthill Campus, Edinburgh Napier University, Edinburgh, United Kingdom; ^5^Cancer Research UK Edinburgh Centre and Division of Pathology Laboratories, Institute of Genetics and Molecular Medicine, University of Edinburgh, Edinburgh, United Kingdom

**Keywords:** biomarkers of radiosensitivity, breast cancer, precision medicine, molecular signatures of radiosensitivity, biomarkers of radiation-induced toxicity

## Abstract

Radiotherapy remains an important treatment modality in nearly two thirds of all cancers, including the primary curative or palliative treatment of breast cancer. Unfortunately, largely due to tumor heterogeneity, tumor radiotherapy response rates can vary significantly, even between patients diagnosed with the same tumor type. Although in recent years significant technological advances have been made in the way radiation can be precisely delivered to tumors, it is proving more difficult to personalize radiotherapy regimens based on cancer biology. Biomarkers that provide prognostic or predictive information regarding a tumor's intrinsic radiosensitivity or its response to treatment could prove valuable in helping to personalize radiation dosing, enabling clinicians to make decisions between different treatment options whilst avoiding radiation-induced toxicity in patients unlikely to gain therapeutic benefit. Studies have investigated numerous ways in which both patient and tumor radiosensitivities can be assessed. Tumor molecular profiling has been used to develop radiosensitivity gene signatures, while the assessment of specific intracellular or secreted proteins, including circulating tumor cells, exosomes and DNA, has been performed to identify prognostic or predictive biomarkers of radiation response. Finally, the investigation of biomarkers related to radiation-induced toxicity could provide another means by which radiotherapy could become personalized. In this review, we discuss studies that have used these methods to identify or develop prognostic/predictive signatures of radiosensitivity, and how such assays could be used in the future as a means of providing personalized radiotherapy.

## Radiotherapy in Breast Cancer Treatment

In 2018 it was estimated that ~18 million new cancer cases and 10 million cancer-related deaths occurred worldwide (1). If current trends in global population growth continue, combined with the effects of an aging population, these figures are predicted to increase to 20 million new cases and 13 million deaths per year by 2030 (2). Breast cancer (BC) is the most common female cancer, with ~2 million new cases and 0.7 million cancer-related deaths occurring per year (1). Despite the multitude of advances made in both the surgical and systemic treatment of cancer patients, radiotherapy (RT) has a key role in the management of nearly two thirds of all cancers (3).

RT is commonly given to BC patients after surgery. These adjuvant RT treatment plans typically involve the delivery of radiation to the tumor in multiple fractions over a period of several weeks. The most common adjuvant RT fractionation regimen following breast-conserving surgery is 25 fractions of 2 Gy over a 5 week period, or hypofractionated regimens consisting of a total of 40 Gy delivered in 15 fractions over 3 weeks (4). An external beam boost to the tumor bed can also be employed following whole-breast RT or integrated simultaneously with whole-breast irradiation (5) for invasive BCs which have a high risk of local recurrence. This boost procedure typically involves either 10 Gy in 5 fractions or 16 Gy in 8 fractions (6, 7), both delivered over the course of 1 week, or a radiobiologically equivalent dose such as 12 Gy in 4 fractions (8). Local recurrences occur most commonly at the original site of the primary tumor due to remaining microscopic tumor cells left following surgery; the objective of adjuvant and boost RT is to eradicate these tumor cells (7, 9). Accelerated partial breast irradiation (APBI) can be considered as an alternative treatment approach to conventional external beam RT or exclusive hormonal therapy which may be particularly useful for patients with a low-risk of local tumor recurrence or for elderly patients. APBI allows the delivery of higher radiation doses in the area of the tumor bed whilst reducing the dose received by normal breast tissue and adjacent organs at risk. Shorter treatment times and acceptable acute toxicity rates can improve patient quality of life whilst also reducing the total cost of treatment (10–14). Linear accelerators are the most commonly used devices for delivering external beam radiation to patients. In recent years, these machines have undergone significant technology-driven improvements that have culminated in the generation of modern radiation-delivery techniques such as intensity-modulated RT and image-guided RT approaches, which are designed to allow highly conformal and precise distribution of radiation to the tumor. Radiation delivery methods such as “dose painting by numbers” or sculpturing are becoming achievable through the use of these machines, meaning that identified subvolumes can be targeted specifically, limiting the radiation dose received by the nearby normal tissues (15). Although these current radiation planning techniques are largely based on advanced imaging to identify the gross tumor volume, the identification of a biological target volume based on the underlying tumor biology could lead to the development of a more personalized radiation treatment plan.

Of patients diagnosed with BC in England between 2013 and 2014, 63% received curative or palliative RT as part of their primary treatment (16). The Oxford overview shows that adjuvant RT halves the 10-year first risk of recurrence (most of which are local breast recurrences) after breast-conserving surgery and systemic therapy in all risk categories (9) and improves survival in lymph node positive patients receiving adjuvant systemic therapy (17). Studies have suggested that RT can benefit up to 83% of BC patients (18) and that breast-conserving surgery followed by whole-breast RT can provide local control and survival rates equivalent to mastectomy (19–21). These results, combined with the advantages of improved cosmetic outcomes and reduced side effects, have made the incorporation of RT into BC treatment standard practice for a large proportion of patients. Unfortunately, not all BC patients gain therapeutic benefit from RT. Although overall 5-year survival rates after RT are ~80%, it is estimated that 30% of these patients will develop local recurrences or metastatic disease, the majority of whom will die within 5 years (22). Additionally, others in the neoadjuvant and palliative settings may only experience an initial partial response or may not respond at all. Side effects, which can affect a patient's quality of life, can also occur with RT.

A variety of imaging approaches for measuring tumor response to RT during or after treatment have been developed; these typically analyse how often and by how much a tumor shrinks anatomically. The RECIST (Response Evaluation Criteria in Solid Tumors) criteria have been widely adopted and are commonly used in oncology clinical trials. This categorizes the tumor response into complete (disappearance of tumor lesions for at least 4 weeks), partial (tumor diameter reduction of at least 30% for at least 4 weeks), stable (neither progressive disease nor partial response) or progressive (tumor diameter increase of at least 20%). Imaging techniques used to measure these changes in tumor size include X-ray, X-ray computed tomography, magnetic resonance imaging and positron emission tomography (23). However, as many of these techniques measure changes in tumor size alone to assess response to treatment, they ignore the underlying biology that drives the response to radiation. Additionally, analyzing changes in tumor size is a measurable clinical outcome that is seen only towards the end or after the treatment has finished, with patients who fail to respond to treatment initially going undetected. This delay may contribute to tumor progression, impact long-term survival and ultimately delay the initiation of an alternative treatment strategy. Non-responding patients will also be at risk of developing RT-induced side effects for no therapeutic gain. Despite the significant evidence that RT can benefit BC patients as a whole, there are still no clinically validated biomarkers that can be used to predict whether neoadjuvant/adjuvant RT will improve outcomes for individual patients.

## Precision Medicine, Prognostic and Predictive Biomarkers

Precision medicine is defined as the incorporation of disease biomarkers, molecular signatures and phenotypes in combination with patient lifestyle and environment into the prevention, investigation and treatment of diseases (24). Using these criteria, patients can be classified into cohorts according to differences in disease susceptibility, prognosis and likely treatment response rates. To improve clinical outcomes, this type of information can be used to select patients that may require more aggressive treatments and those that are most likely to benefit from specific treatments (25). Genomic instability is a key feature of cancer that is characterized by genetic and epigenetic heterogeneity (26–28); it is therefore not surprising that patients diagnosed with the same cancer type vary in prognosis and in their responses to treatment. As we improve our understanding of the fundamental processes that control carcinogenesis and pathogenesis, precision medicine will become more and more important in the management of cancer patients. An era of personalized cancer medicine, in which biomarkers can be used to tailor treatment to each specific patient, is a major goal in oncology.

Biomarkers can be defined as characteristics which can be evaluated and measured as indicators of normal biological processes, pathogenesis or response to therapy (29). Cancer biomarkers may be diagnostic, prognostic, predictive, or used to monitor treatment responses. Prognostic biomarkers provide information about a patient's overall cancer outcome, irrespective of therapy. They can identify high-risk patients who may benefit from more aggressive treatments but provide no information on which patients will most likely derive a clinical benefit from a specific therapy. Conversely, predictive biomarkers can indicate the probability of a patient gaining a therapeutic benefit from a specific treatment (30–32). These fundamental prognostic and predictive biomarker concepts have been integrated into the precision medicine initiative.

BC radiomics, an emerging field in precision medicine, is the process of extracting quantitative information from medical images to influence patient treatment. This concept assumes that extracted imaging data are the result of biological processes occurring at a genetic and molecular level, and are therefore associated with the genotypic and phenotypic characteristics of the tumor (33). Radiomic features have been correlated with BC clinical data including stage, lymph node involvement and hormone receptor status (34), and also have the ability to discriminate between malignant and benign lesions (35). While radiomics has the potential to contribute to BC precision medicine in the future, BC molecular classification systems based on microarray gene expression analysis are currently being used clinically. These classification systems have identified several intrinsic BC molecular subtypes which have been shown to differ in treatment responses and predict disease-free survival and overall survival (36–39). Unfortunately, microarray analysis or genome sequencing for individual patients is cost-prohibitive for routine clinical use. To overcome this issue, studies have investigated the potential utility of using smaller gene sets to stratify BC patients, providing prognostic and/or predictive information which can be employed by clinicians to guide the use of adjuvant chemotherapy or endocrine therapy (40). These clinically-available tests include the breast cancer index (41), Endopredict (42), the Oncotype DX 21-gene recurrence score (43), the BreastOncPx 14-gene distant metastasis signature (44), and the MammaPrint 70-gene prognosis signature (45). Finally, a 50-gene signature called PAM50 (now commercialized under the name Prosigna) has improved the ability to predict recurrence of estrogen receptor^+^/lymph node^−^ BC patients compared to models using only clinical variables (46, 47). Although these clinically validated genetic tests have made significant changes in the way patients are selected for receiving chemotherapy or endocrine therapy, RT treatment plans still rely upon historically standardized, one-size-fits-all therapeutic approaches.

**Figure 1:**
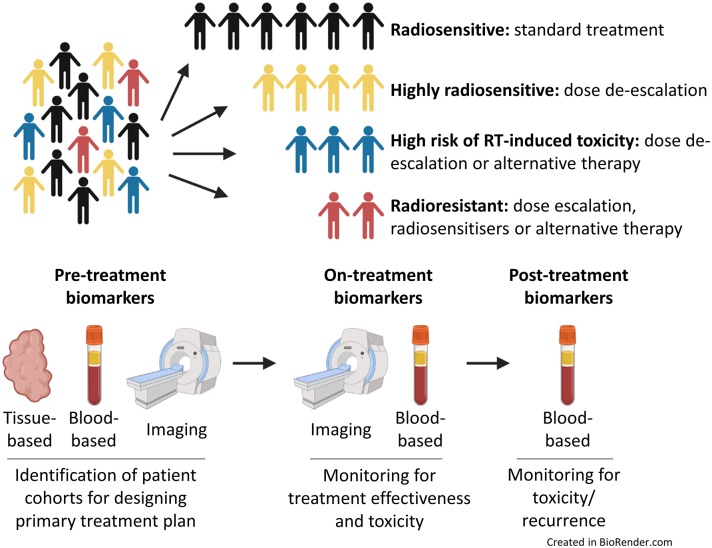
Precision medicine and radiotherapy. Patients could be stratified into different cohorts based on predicted intrinsic radiosensitivity and risk of toxicity. On-treatment monitoring may provide information on response to treatment, enabling adaptive changes to a patient's treatment to be made if necessary. Post-treatment biomarkers could be used to assess for evidence of toxicity, tumor recurrence or the development of metastatic disease.

A move toward more personalized RT treatment, tailored to individual risk and tumor biology, would help improve patient outcomes (48). Many factors are known to influence a tumor's response to irradiation, including total dose, fractionation, tumor doubling time, hypoxia and intrinsic radiosensitivity. If RT is to become part of the precision medicine initiative, we need to identify clinically-validated biomarkers that can predict response to RT before starting treatment or biomarkers that can help clinicians assess a tumor's response to RT during treatment (49). Currently there are no sufficiently validated biomarkers of radiosensitivity for routine application in the clinic (49). These assays/biomarkers might allow the tailoring of radiation dose regimens to individual patients based on tumor biology or the prediction of the risk of localized tissue toxicity. Patients unlikely to benefit from RT could be spared radiation-induced toxicities and associated co-morbidities, whilst allowing more effective therapies to be instigated at an earlier stage of the treatment process (Figure 1). As discussed above, molecular signatures have been developed that enable clinicians to more individually tailor systemic therapies for patients with BC. Slower progress is being made to develop tools to predict RT response; in part this is due to radiation oncology vendors not having the financial capacity to support genomic-orientated collaborations with radiation oncology investigators (50). Nonetheless, much work has been performed to try and develop such biomarkers. The aim of this review is to highlight recent developments in this exciting yet under-researched field.

## Molecular Signatures of Radiosensitivity

### Intrinsic Subtypes

Gene expression profiling and histopathological classification using the expression of estrogen receptor (ER), progesterone receptor (PgR) and human epidermal growth factor receptor 2 (HER2) can be used to classify BC into different subtypes. These subtypes can be broadly categorized into luminal, normal-like, HER2-overexpressing and triple negative breast cancer (TNBC) (36–39, 51). Gene expression profiling can stratify BC subtypes to a greater degree than histological assessment; however, as previously mentioned, this is often not feasible for use with large scale patient populations due to financial constraints (52). Both classification methods have confirmed the heterogeneous nature of BC which can lead to substantial differences in biology, pathogenesis, treatment response and patient outcome (39, 53, 54). Luminal subtypes (which correspond to ER^+^ BC) are associated with a more favorable prognosis, whereas HER2-overexpressing and TNBC subtypes are associated with significantly worse recurrence-free survival and overall survival.

BC subtyping has also been investigated for its use in predicting response to RT. The major advantage of using these subtyping markers in the selection of patients for RT is that testing for these markers using biopsy samples is now mandatory in the clinics, as they are used for selecting patients for hormonal or targeted therapies. *In vitro* studies using BC cell lines have shown that individual subtypes exhibit differential inherent sensitivities to radiation (55). Multiple clinical analyses have also shown that subtype is related to radiosensitivity; one large study reported that local recurrence for invasive BC treated with breast-conserving surgery followed by RT was 0.8% for luminal A, 1.5% for luminal B, 8.4% for HER2-overexpressing and 7.1% for TNBC (56). HER2-overexpressing and TNBC have also been associated with an increased risk of local recurrence and distant metastasis in combination with reduced overall survival in patients treated with post-mastectomy RT or RT alone (56–58). Improved overall survival after post-mastectomy RT has also been identified in ER^+^/ PgR^+^/HER2^−^ patients, whereas no significant overall survival improvement was seen following the same treatment in ER^−^/ PgR^−^/HER2^+^ patients (58). Similar results were observed when using the Oncotype DX recurrence score to predict overall survival following post-mastectomy RT; this study suggested that low-risk patients, as determined by low OncotypeDX recurrence scores, had significantly improved overall survival following post-mastectomy RT compared to those low-risk patients that did not receive this treatment. In comparison, post-mastectomy RT was not of significant benefit to intermediate and high-risk patients. The authors suggested that OncotypeDX recurrence score may be a predictor of survival benefit from post-mastectomy RT (59). Furthermore, improved overall survival has been documented in patients with ER^+^/ PgR^+^/HER2^−^ tumor who received post-mastectomy RT when compared with those who received no RT (58). This suggests that RT is particularly effective for breast cancers of the luminal phenotype.

### Pan-Cancer Genomic Signatures

The first pan-cancer genomic radiosensitivity signature was developed using 35 cancer cell lines from the National Cancer Institute-60 (NCI-60) panel (60). Torres-Roca et al. (60) used gene expression data combined with the survival fraction of cells that received a dose of 2 Gy (SF2), an accepted experimental measure of cellular radiosensitivity, to develop a radiation classifier that could predict inherent radiosensitivity. Their results showed that the classifier successfully predicted SF2 values in 22 of 35 NCI-60 cell lines. The authors then went on to identify three novel genes (*RbAp48, RGS19*, and *R5PIA*) whose expression was correlated with radiation sensitivity. These results were the first to show that gene expression profiles had the potential to predict radiation sensitivity and that genomics could be used to identify novel radiosensitivity molecular markers. Unfortunately, subsequent studies have failed to reproduce these *in vitro* results using modern gene expression techniques; therefore, the validity of this signatures use in cell lines remains open to debate (61).

This pan-cancer genomic radiosensitivity signature has since been developed by the same group to include biological variables such as tissue of origin, p53 and ras status, known influencers of radiosensitivity. Using SF2 values from 48 cancer cell lines from the NCI-60 panel, gene expression analysis was performed to identify a 10 gene signature associated with intrinsic radiosensitivity (*AR, cJun, STAT1, PKC, RelA, cABL, SUMO1, CDK1, HDAC1*, and *IRF1*). These 10 genes are associated with specific pathways that included cell cycle, DNA damage response, histone deacetylation, proliferation and apoptosis. These results were used to produce a radiosensitivity index (RSI), whereby lower RSI correlates with greater radiosensitivity (62). The RSI has been used in clinical studies; these have shown the signature to be disease-site independent, predicting clinical outcomes in esophageal, rectal, head and neck, prostate, pancreas, colon, glioblastoma and non-small cell lung cancer patients following RT (62–66). However, some of these results were obtained from pilot studies which had low patient numbers, whereas others did not compare the results with non-RT treated controls. The RSI has also been evaluated in 2 independent BC patient cohorts. Results indicated that RT-treated patients classified as radiosensitive by RSI had improved recurrence-free survival or distant metastasis-free survival at 5 years, but there was no difference in recurrence-free survival or distant metastasis-free survival between radiosensitive and radioresistant patients in those not treated with RT. The authors suggested that RSI is RT-specific, because it was not prognostic for patients treated with surgery alone, but importantly can be used as a predictive signature of RT benefit. Furthermore, they demonstrated that the impact of RSI was affected by ER status, with RSI-classified radiosensitive patients having greater distant metastasis-free survival in ER^+^ patients (67). RSI has also been integrated with BC molecular subtyping to predict local recurrence; Torres-Roca et al. (68) showed that although RSI did not uniformly predict for local recurrence, it was able to identify a subpopulation of TNBC that had high RSI scores which were classified as radioresistant with the highest risk of local recurrence. They also illustrated that RSI could identify a luminal radioresistant subpopulation that would benefit from radiation dose escalation. They concluded that the combined use of RSI and molecular subtyping could help guide the selection of patients for RT treatment in BC (68).

In an attempt to tailor radiation doses across differing BC subtypes, researchers have integrated the RSI with the linear quadratic model to derive a genomic-adjusted radiation dose (GARD); this aimed to predict which tumors would gain an enhanced therapeutic effect from RT. GARD values were calculated for over 800 tumor samples using data from the prospective, observational Total Cancer Care cohort. The results demonstrated that there was a wide range of GARD values within tumor types and that GARD values could independently predict clinical outcome in lung, pancreatic, glioblastoma and BC patients (69). The limitations of this study included the failure to evaluate the prognostic potential of GARD (no data were evaluated for patients that did not receive RT) and a lack of external validation. However, this GARD-based clinical model could allow for RT personalisation based on radiation dose tailored to tumor radiosensitivity and may provide a means to develop genomically-guided RT-based clinical trials.

A similar approach which used the same NCI-60 cell line panel was undertaken to identify not only genes whose expression profiles were related to intrinsic radiosensitivity, but also genes whose expression changed following radiation treatment and were associated with post-radiation survival. Changes in gene expression induced by radiation were found to be similar between different tumor types and were also associated with p53 status. The authors suggested that there was possibly a conserved set of genes responsible for a specific radiation response (70). However, this work contradicts a more comprehensive study that profiled the radiation response of over 500 cell lines which showed sensitivity to radiation was characterized by significant genetic variation within and between cell line lineages. As well as identifying genes whose expression was associated with response, they also identified somatic copy number alterations and gene mutations that correlated with post-radiation survival (71).

In addition to radiation-induced gene response signatures, other groups have looked at signatures that may predict response to radiation treatment for a range of different cancer types. These gene expression profiles include hypoxia-related signatures (72–74), cell cycle and DNA damage gene-related signatures (75, 76), along with signatures predicting response to radiosensitising drugs (77, 78). As with many of the other *in vitro* derived signatures, none of these has thus far withstood stringent external validation and therefore have yet to be translated into clinical practice.

### Breast Cancer Specific Genomic Signatures

With a technique similar to that employed to produce the RSI, a different study used only BC cell lines to develop a BC specific radiation sensitivity signature (RSS) (79). Their aim was to produce a gene signature that could predict the radiation response of BC patients and allow the identification of patients with tumors refractive to conventional RT regimens. To derive their gene signature, intrinsic radiosensitivity was correlated with gene expression using SF2 values from a panel of 16 BC cell lines. Interestingly, Speers et al. (79) found no association between intrinsic radiosensitivity of the BC cell lines and subtype classification, contradicting findings from previous publications. A 51 gene signature, enriched for pathways involved in DNA damage response and cell cycle, was developed from their results. Validation of this gene signature, the most promising to date, was performed in two independent clinical BC datasets in which patients had been treated with breast-conserving surgery and RT. The results showed that the RSS could provide information on which patients were likely to respond poorly to standard RT regimens (79). However, as all patients in the studied cohorts received standard RT, the 51 gene signature could not be validated for its predictive potential. This RSS has subsequently been marketed as RadiotypeDX and is regarded as having potentially similar applications to that of OncotypeDX, which is used for selecting the most appropriate systemic therapies for BC patients. RadiotypeDX is currently undergoing external validation, using tissue and clinical data from a randomized controlled trial evaluating post-operative RT after breast-conserving surgery in patients with early BC, who received appropriate adjuvant systemic therapy according to ER status (80). Although the results are yet to be published, these types of trials, as well as prospective randomized trials or “prospective retrospective” analyses from phase III trials, are essential in order to show that these kinds of signatures have clinical applications and that they could be integrated into clinical practice (81).

More recently a refined version of RadiotypeDX (Adjuvant Radiotherapy Intensification Classifier [ARTIC]) has been validated in a Swedish Conservation trial in which patients were randomized to post-operative whole-breast RT or no RT (82). Of note, a limited number of patients in this trial received systemic therapy. ARTIC was found to be highly prognostic for loco-regional recurrence and predictive of benefit from RT. Patients who had a low ARTIC score derived a substantial benefit from RT; in contrast, patients with high ARTIC scores had less benefit from RT. The authors recognized the requirement to validate ARTIC within a trial of patients treated by breast-conserving therapy, systemic therapy +/– post-operative whole-breast irradiation.

A gene profile called DBCG-RT, predictive for therapeutic benefit from post-mastectomy RT and prognostic for locoregional control, was developed by the Danish Breast Cancer Cooperative Group and has been independently validated (83). The DBCG-RT gene profile can divide patients into those with a high- and low-risk of local recurrence and can identify a subcategory of low-risk patients who obtain no additional benefit from RT. A correlation between patients that were at an increased risk of developing local recurrence and non-luminal, ER^−^ tumors (basal and HER2-overexpressing) was observed, while the low local recurrence risk group correlated with the luminal A subtype. These findings indicate that intrinsic subtyping and the DBCG-RT profile can identify the same tumor types and their responses to RT treatment.

Others have attempted to develop specific radiation signatures for BC that distinguish patients who require treatment intensification, for whom traditional therapies (surgery, chemotherapy and RT) are inadequate. Gene analysis has been conducted on early stage BC patients, all treated with RT after surgery, to try and distinguish gene signatures prognostic of local recurrence in patients treated with radiation. A radiation signature consisting of 81 genes outperformed pathologic and clinical characteristics for predicting local recurrence (84). A similar study performed gene expression microarray profiling to identify differentially expressed genes between tumors from patients who developed local recurrence after breast-conserving surgery and radiation, and patients who did not; Kreike et al. (85) derived a 111 gene signature, enriched for cell proliferation, which was independently associated with local recurrence. Unfortunately, both of these signatures failed external validation.

The Early Breast Cancer Trialists' Collaborative Group meta-analysis suggested that most early stage BC patients treated with breast-conserving surgery are cured of their disease with both surgery and endocrine therapy alone, without the need for RT (9). Studies have also suggested that RT may be omitted in selected elderly patients with low-risk disease (86). RT omission after breast-conserving surgery has also been explored in several randomized phase III trials; unfortunately, heterogeneous eligibility criteria across the trials has resulted in differing results with confounding interpretations (87). These types of studies have led to an increased interest in the development of radiation omission signatures specific for BC, whereby low-risk patients can be spared RT. An omission signature has yet to be developed; however, different groups are attempting to validate a variety of previously described molecular classifiers for this purpose. Trials are ongoing to assess the potential of OncotypeDx (IDEA trial), Prosigna (PRECISION trial), IHC (LUMINA trial), and IHC4 (PRIMETIME trial) scores as stratification methods for radiation omission (81).

### Barriers to Clinical Adoption of Molecular Signatures

Although many molecular signatures have been developed to predict a tumor's response to radiation, most have failed external validation and, as a result, none have gained approval for clinical use. In part, as indicated earlier, the barriers are the high costs of academic-industry collaborations and in part scientific (50). Unfortunately, the gene profiles derived from the studies outlined in this review show little similarity to each other, which suggests that the methods used to produce the signatures may influence which genes and pathways are selected. Like many other cancers, BC is a complex heterogenous disease; differences in pathway activation can lead to different drivers of oncogenesis, even within the same subtype. These significant differences will influence how each tumor reacts to RT; it is possible that gene signatures are unable to account for the complexity of a tumor's radiation response. These issues are likely to be more pronounced in profiles derived from cell lines, as these are clonal populations that cannot account for tumor heterogeneity and the effects of the tumor microenvironment. Validation of the signatures using clinical trial data is also complicated; variations between trials in terms of the radiation treatment regimens used, inclusion of different patient subtypes and inconsistencies between the numbers of treated and control patients included in the studies makes the generation of meaningful data and rigid signature validation challenging. The methods used for RNA extraction and subsequent gene expression analysis can also differ between the original studies which developed the profiles and the clinical trials in which they are being validated. These issues are specific for gene signature validation; there are also more general hurdles that must be overcome if lab-based research is to be successfully translated into a clinically applicable test. These include the development of standard operating procedures and making tests cost effective and easy to use, while also providing evidence that they can improve upon standard practices already in place in the clinics. The combination of these gene signature specific and general issues are factors that are currently contributing to their lack of clinical translation and use in the clinic.

## Gene Mutations, mRNA and Intracellular Protein Markers

Several studies have looked at individual biomarkers, rather than cohorts of genes, for their potential to correlate with a tumor's response to RT. In BC, expression levels of Holliday junction recognition protein mRNA can be prognostic for disease-free survival and overall survival, thereby predicting patient sensitivity to RT (88). High cytoplasmic expression of peroxiredoxin-I has also been shown to correlate with increased local recurrence after RT (89). Proteasome (prosome, macropain) 26S subunit, non-ATPase, 9 (PSMD9) is another protein whose elevated expression was associated with increased incidence of local recurrence in a cohort of patients that received adjuvant RT, but not in those that did not receive RT; the authors concluded that this protein might therefore be a predictive biomarker for RT response (52).

The *BRCA1/2* genes are part of the granin gene family and function as tumor suppressors; they play critical roles in maintaining genome stability through controlling pathways involved in DNA damage response/repair, cell cycle and transcription (90). Previous studies have shown that *BRCA1/2* gene deregulation is associated with BC carcinogenesis (91), with germline *BRCA1/2* mutations accounting for up to 10% of all cases (92). Inherited *BRCA1/2* mutations are estimated to increase the risk of BC by to 84% (93–95). These tumors exhibit ineffective homologous recombination DNA repair, causing an accumulation of genetic mutations, which can drive carcinogenesis. Although tumors deficient in a DNA repair mechanism may be expected to exhibit radiation sensitivity, they are instead thought to rely upon alternative DNA repair mechanisms that are more effective at repairing radiation-induced DNA damage. PARP enzymes play a key role in the repair of DNA single-strand breaks through the repair of base excisions, a process normally performed by error-free homologous recombination (96). The inhibition of PARPs can result in the accumulation of DNA single-strand breaks, leading to the generation of double-strand breaks at replication forks. Double-strand breaks are usually repaired by error-free homologous recombination, a process that is inhibited in BRCA1/2 mutated tumors; these double-strand breaks can lead to apoptosis of cancer cells within the tumor. The PARP enzyme inhibitor olaparib is currently undergoing trials to assess its clinical usefulness and cost effectiveness in the treatment of BRCA1/2 mutated HER2^−^ metastatic BC patients following chemotherapy (97). This drug has previously gained clinical approval for use in patients with recurrent, platinum-sensitive, BRCA1/2 mutated ovarian cancers (98). Furthermore, the small molecular PARP inhibitor niraparib has been shown to radiosensitise a variety of human xenograft tumors, including the triple-negative MDA-MB-231 human BC cell line (99). It has therefore been suggested that BRCA status could be a useful biomarker for stratifying patients for the use of PARP inhibitors in combination with RT.

## Blood-Associated Biomarkers of Tumor Radiosensitivity

The identification of circulating prognostic or predictive biomarkers in the blood has advantages over tissue-based approaches as it is non-invasive and does not require a biopsy. These liquid biopsies can be taken pre-, post- or on-treatment, allowing for continual patient monitoring with the potential to assess the tumors response to treatment. These biomarkers could hold particular promise in patients with metastatic disease, where monitoring for progression is critical but where repeated biopsy sampling is often unfeasible due to the location of the lesions. Studies have investigated the use of blood based biomarkers, such as carcinoembryonic antigen and carbohydrate antigen 15–3 for primary diagnosis and the detection of metastatic disease (100–105), with others examining the correlation between serum HER2 concentration and tumor HER2 status (106–109). Although the use of blood-based biomarkers to assess tumor pre-treatment radiosensitivity or on-treatment response to RT has been less intensively studied, there is a growing interest in the use of these types of biomarkers for precision RT. Areas of research currently under investigation include exosomes and circulating tumor cells (CTCs).

Exosomes are formed from the inward budding of the membrane of multi-vesicular bodies and are ~40–100 nm in diameter. The contents of exosomes are released from cells via endocytosis through fusion of their membrane with that of the cell's plasma membrane. Exosomes have been shown to contain nucleic acids, proteins, lipids and enzymes (110). There is increasing evidence that exosomes play roles in tumorigenesis and cancer progression, including immune suppression, angiogenesis, cell migration and invasion (111–115); as a result, their use as liquid biopsy biomarkers through exosomal profiling is being investigated for disease diagnosis and therapy efficacy monitoring. The transfer of exosome contents to local tumor or stromal cells in the tumor microenvironment, or to a distant site within the body, has been shown to be a mechanism through which cancer cells can transmit the malignant phenotype to normal cells and establish a suitable environment for metastatic colonization (113). Exosomes transferred from stromal to BC cells can also contribute to chemotherapy and radiotherapy resistance. Resistance mechanisms mediated by exosomal transfer are thought to involve anti-viral and NOTCH3 pathways (116). Tumor oxygen concentrations at the time of radiation have been shown to influence cell radiosensitivity (117). When exposed to a given dose of radiation, cancer cells in low oxygen states can withstand 2–3 times higher doses than aerobic cells. Known as the oxygen enhancement effect, the role that oxygen plays in RT is described through the oxygen fixation hypothesis (118, 119). Oxygen present at the time of RT can react with radiation-induced DNA radicals producing permanent DNA damage; however, in the absence of oxygen this damage can be repaired by free radical scavengers such as endogenous thiols (120), giving these hypoxic cells a significant survival advantage. Hypoxia leads to the increased production of exosomes (121, 122), with the transfer of exosomes from stromal to BC cells stimulating signaling pathways related to radiation resistance (116). The tumor-associated exosome profile may also give an indication of the oxygenation status of breast tumors, and therefore could be used to identify radioresistant tumors (122).

CTCs and circulating tumor DNA are cells or DNA that have been shed by the tumor into the systemic circulation; these can be indicators of residual disease following treatment and are likely to represent an important mechanism through which a tumor can metastasise (123, 124). CTCs have been detected in up to 30% of non-metastatic BC patients and their presence (even just one CTC) has been shown to be prognostic for recurrence-free survival and overall survival (125–135). CTC detection is also related to metastasis and poor survival in low-risk patients with lymph node negative disease who did not receive adjuvant therapy (133). Data from the National Cancer Database and SUCCESS (Simultaneous Study of Gemcitabine-Docetaxel Combination Adjuvant Treatment as well as Extended Bisphosphonate and Surveillance) clinical trials have been used to investigate the use of CTC status to predict the benefit of RT in early stage BC (136). The results suggested that CTC status could predict the effectiveness of RT and showed that CTC^+^ patients had improved local recurrence-free survival and disease-free survival following RT, whereas CTC^−^ patients obtained no benefit. Furthermore, CTC^+^ patients that received RT had improved overall survival compared with CTC^+^ patients who did not receive RT. Pooled analysis using both cohorts who underwent breast-conserving surgery indicated that RT was associated with longer overall survival in CTC^+^ patients, but not in CTC^−^ patients. However, CTC status was not associated with an overall survival benefit in patients who underwent mastectomy and RT. Overall, these results suggested that CTC status may be used as a predictive marker for assessing the potential benefit from incorporating RT into the treatment of early stage BC for patients undergoing breast-conserving surgery (136, 137).

Although circulating tumor biomarkers have the potential to be used for cancer diagnosis, measuring response to treatment and monitoring for recurrence, studies evaluating their clinical usefulness are still limited in number. While advances have been made in this field, circulating tumor biomarkers in BC patients are not yet employed for primary diagnosis, largely due to their low sensitivity and specificity, and also because of a lack of validation through large prospective trials. Such trials are essential to fully evaluate their prognostic or predictive potential in determining tumor radiosensitivity and to enable their use in the future (138).

## Biomarkers of Radiation-Induced Toxicity

In contrast to the relatively limited research involving blood-based biomarkers for tumor radiosensitivity, the use of circulating biomarkers to assess radiation-induced toxicities has attracted much greater interest. Like any cancer treatment, RT has the potential to cause toxic side effects in normal tissues; these ultimately dictate the total dose that can be delivered to a patient, which can influence outcome. In BC, radiation-associated side effects include cardiac and skin toxicities, fibrosis, lymphedema, secondary cancers, rib fractures and brachial plexopathy (139). In some studies the combination of tamoxifen and RT has resulted in an increased risk of fibrosis, hypothesized to be due to a tamoxifen-induced increase in TGF-β (140–144). Even with highly conformal intensity-modulated RT and image-guided RT, which aim to spare regional organs from radiation exposure, these side effects can occur in up to 15% of patients and can seriously affect patients' physiological and physical quality of life (145, 146). Taking into account the high survival rate of BC patients, there is a need to develop tests that can be used either pre-treatment, or at an early stage of the treatment process, to predict which patients are at a higher risk of developing radiation-associated side effects (147). Predicting radiation-induced toxicity could enable better treatment regimens to be devised for individual patients in a range of cancer types. Radiation toxicity is related to treatment schedule (dose and duration), patient specific factors and genetic factors. With these in mind, several tests have been proposed to classify individual patient radiosensitivity based on the induction of DNA damage and radiation-induced apoptosis, in addition to gene expression profiles (148).

One area of research that holds promise in predicting the risk of late radiation-associated toxicity is through assessment of the radiation-induced initial DNA damage response of peripheral blood lymphocytes through DNA double strand break quantification, comet or micronucleus assays (148). A relationship between high rates of double strand breaks in peripheral blood lymphocytes and late grade 3 skin and subcutaneous tissue toxicities in BC patients has suggested that assessing the initial DNA damage could be useful for predicting radiation toxicity (149). Unfortunately, various studies assessing the radiation-induced DNA damage through other methods have shown both negative (150, 151) and positive (152–154) associations with radiation toxicity. Additionally, the clinical usefulness of radiation-induced DNA damage response assays is yet to be fully determined. Determining peripheral blood lymphocyte radiation-induced apoptosis appears to be the most promising assay for determining radiation toxicity. Studies have shown that radiation-induced apoptosis increases with higher doses of radiation; however, elevated levels of radiation-induced apoptosis were associated with reduced risk of late radiation toxicities (155). In BC patients, studies have used a combination of DNA damage assessment and levels of radiation-induced apoptosis to assess radiation-induced toxicity. Henríquez-Hernández et al. (156) demonstrated that patients showing lower levels of initial DNA damage and higher levels of radiation-induced apoptosis were considered resistant to RT. These patients were at a lower risk of suffering severe subcutaneous late toxicities after treatment with high radiation doses. Although these results were based on a small number of patients, it provided evidence that dose escalation can be achieved in patients that are resistant and tolerant to higher radiation doses.

There is an association between the dose of radiation received by the heart and increased risk of heart disease (157–159); as the dose increases so does the risk of developing heart complications. Cardiovascular side effects are a major concern for clinicians treating BC patients with RT (160, 161), especially for left sided BC or patients with pre-existing cardiovascular disease. Cardiotoxic effects of RT can be seen for several years after RT, and increased risk may remain for at least 2 years post-RT (161). Pericarditis, valvular dysfunction, cardiomyopathy and coronary artery disease are some of the severe late cardiotoxic effects seen in up to 30% of BC cases within 5–10 years following RT (162). Blood biomarkers have been investigated for their roles in detecting and monitoring cardiotoxicity and assessing early signs of cardiovascular dysfunction following RT. Brain natriuretic peptide and its amino terminal fragment are biomarkers produced by ventricular myocytes, with elevated blood levels commonly seen in unstable angina, myocardial infarction and cardiac failure (163). Several studies have shown that levels of these biomarkers increase after RT, suggesting that these may be also be biomarkers of early radiation-induced cardiotoxicity (164–166). Circulating cardiac troponin I and cardiac troponin T are highly sensitive and specific biomarkers for cardiac disease and have been shown to be useful for detecting cardiotoxicity following chemotherapy (167, 168) and, although some studies indicate they do not increase following RT (165, 169), others show that they do (164, 170, 171). While these traditional cardiac biomarkers are the most extensively studied for cardiotoxicity following RT, other blood biomarkers that have been investigated include C-reactive protein and lipopolysaccharide. C-reactive protein, an acute phase protein whose expression is related to inflammatory cytokines, has been proposed as a biomarker of radiation-associated cytotoxicity as studies have highlighted an association between elevated C-reactive protein levels and adverse prognosis in patients with heart failure (172–174). Although Lipshultz et al. (166) found increased levels of C-reactive protein in children following treatment for a variety of cancers with chemotherapy and RT, the majority of studies have failed to find an association between circulating C-reactive protein levels and myocardial damage post-chemotherapy (175–177) or RT (178). Lipopolysaccharide-binding protein has been investigated as a potential biomarker for lung toxicity after chest RT (179). In BC, lipopolysaccharide-binding protein concentrations were observed to increase within 24 h post-RT and remained elevated 1 month after RT. Raised lipopolysaccharide-binding protein levels were also correlated with cardiac dysfunction, which was evaluated up to 3 years following the completion of RT, suggesting that lipopolysaccharide-binding protein could be a potentially useful prognostic biomarker of RT-induced cardiotoxicity (178). Characteristics such as early detection in the blood and persistent kinetics, in combination with concentrations being related to toxicity, have made lipopolysaccharide-binding protein an attractive biomarker for clinical use. Other proposed biomarkers include heart-type fatty acid-binding protein, glycogen phosphorylase isoenzyme BB, myeloperoxidase and nitric oxide (180). Although these biomarkers have yet to be used clinically, they could in the future play key roles in determining which RT patients may require dose de-escalation, or even the provision of alternative treatments if BC patients can be classified as high-risk for developing radiation-induced side effects post-treatment.

**Figure 2:**
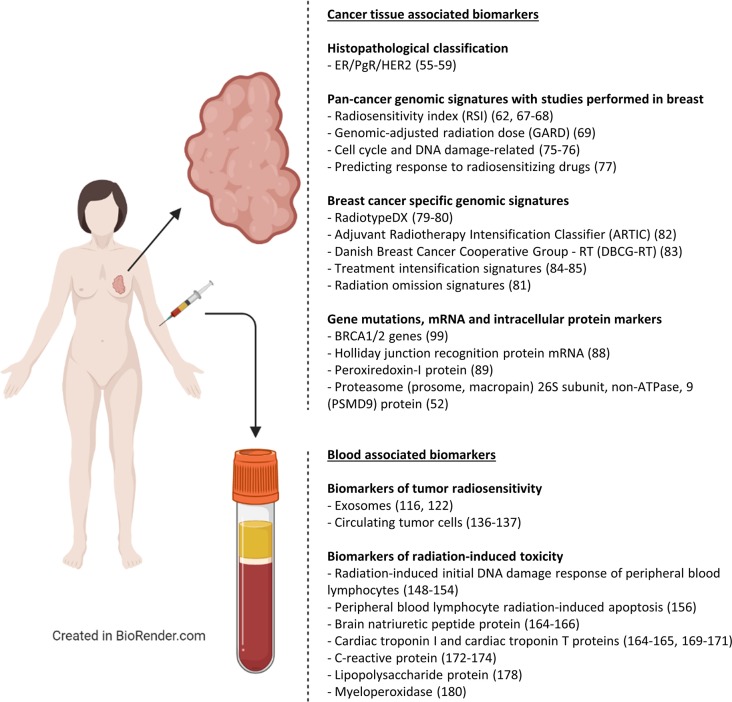
Biomarkers of radiotherapy response for breast cancer. An overview of cancer tissue-associated and blood-associated biomarkers of radiotherapy response that have been developed for breast cancer patients (ER, Estrogen receptor; PgR, Progesterone receptor; HER2, human epidermal growth factor receptor 2).

A summary of the techniques used to identify biomarkers of radiosensitivity that have been described in this paper is provided in Figure 2 and Table 1. Table 1 also outlines the specific advantages and disadvantages associated with each method.

**Table 1:** Techniques used for measuring the response of tumors to RT.

**Imaging-based methods**
Method: Advanced imaging modalities can provide information on tumor size (X-ray computed tomography, ultrasound, magnetic resonance imaging) and give an estimation of hypoxia and proliferation heterogeneity within different areas of a tumor (positron emission tomography). Imaging can be conducted pre-, during and post-treatment. Advantages: Non-invasive. Real-time measurements of response can be obtained. Methods, protocols and criteria for visualizing and assessing changes in tumor size are already established within the clinic. Disadvantages: Changes in tumor size can be gradual and slow which may only be seen towards the end or after the treatment has finished; patients who fail to respond will initially go undetected. Patient safety concerns over repeat exposure to radiation and radioisotopes.
**Cancer tissue-based biomarkers**
Method: Evaluating the expression levels of genes or proteins using biopsy samples. Advantages: Pre-treatment biopsies are already taken as part of standard of care treatment, meaning patients do not require an additional procedure. Protein assessment using IHC can identify the location of protein expression and provide information on functional status. Gene signatures may be prognostic and predictive of responses to RT. Disadvantages: Invasive. Unable to monitor response to treatment without further invasive procedures, which can be difficult to obtain and hard to justify. Some methods of analysis are cost prohibitive. Does not provide real-time measurements of response.
**Blood-based biomarkers**
Method: Measuring the presence or expression levels of CTCs or proteins using liquid biopsy (blood) samples. Samples can be obtained readily pre-, during and post-treatment. Advantages: Non-invasive. Real-time measurements of response can be obtained. Disadvantages: Low sensitivity and specificity of tests developed to date.

*Advantages and disadvantages of each method are provided (IHC, immunohistochemistry; RT, radiotherapy; CTCs, circulating tumor cells)*.

## Conclusion

Significant advances have been made in the development of molecular signatures to stratify BC patients for more personalized targeted and endocrine therapies; however, similar improvements in the field of personalized RT have yet to be adopted in the clinic. Technological developments in the methods used for radiation delivery have allowed radiation oncologists to accurately conform radiation to the tumor; however, only limited methods of analyzing response to treatment are available. To truly achieve personalized RT, we also need to be able to stratify patient's pre-treatment based on individual patient radiosensitivity and through analyzing the tumor's response to RT during treatment. Unfortunately, there are currently no clinically-validated prognostic or predictive signatures/biomarkers that can reliably classify patients into those that would benefit the most from RT, those that could be safely treated with dose escalation or de-escalation, or those that should be treated without RT. While preliminary efforts to develop these signatures/biomarkers have been encouraging, there is still much work to do in order to refine and validate them. Ultimately, for any of these tests to be translated into the clinic, studies will need to demonstrate their accuracy and reproducibility, and perhaps more importantly, exhibit their utility in improving outcomes or refining the selection of patients for RT through clinical trials. While not yet realized, the ongoing development of these signatures and biomarkers holds much promise; the linking of these signatures and biomarkers with other techniques, such as imaging, would help deliver an overall precision medicine package that could greatly enhance the effectiveness of RT. There is confidence within the scientific community that personalized medicine will finally be realized for BC patients undergoing radiation treatment in the decades to come.

## Author Contributions

JM, MG, DA, and AT conceptualized the article. JM wrote the majority of the manuscript. Figures and tables were composed by JM and MG. Critical revisions were made by JM, MG, CM-P, CK, LP, JAF, AVP, IK, SPL, DA, and AT. All authors read and approved the final manuscript.

## Conflict of Interest

The authors declare that the research was conducted in the absence of any commercial or financial relationships that could be construed as a potential conflict of interest.
